# Current Status of Childhood Obesity and its Associated Morbidities in Turkey

**DOI:** 10.4274/jcrpe.506

**Published:** 2012-03-08

**Authors:** Abdullah Bereket, Zeynep Atay

**Affiliations:** 1 Marmara University, Department of Pediatrics, Division of Pediatric Endocrinology, Istanbul; +90 216 625 45 45/9109+90 216 411 60 49 abdullahbereket@gmail.comAbdullah Bereket MD, Marmara University, Dr Faruk Ayanoğlu Street No: 50/11, Fenerbahce, İstanbul, Turkey

**Keywords:** childhood, obesity, current status

## Abstract

As a transitional society, rapid changes have occurred in the social, economic, nutritional and lifestyle aspects of the Turkish population over the last three decades. As a result, the prevalence of overweight and obesity has shown a dramatic increase in the adult Turkish population, reaching figures as high as 30-40%. Although there is no nationwide figure regarding the prevalence of overweight and obesity in Turkish children and adolescents, several local studies performed between 2000 and 2010 in different regions of the country have demonstrated varying prevalence rates of 10.3%-17.6% and 1.9%-7.8% for overweight and obesity, respectively, in children aged 6-16 years. The differences in the figures obtained in these regions are thought to be due to variations in the subject sampling. The figures appear to vary depending on residential (urban vs. rural) and economic conditions. Belonging to a high-income family, living in a large city, having obese parents, being of high birthweight, consuming sugar-sweetened beverages (soft drinks, juice drinks, etc.), and spending time in front of TV and PC were identified as the most common risk factors. Complications and co-morbidities of obesity have also started to appear in our pediatric population. Metabolic syndrome, diagnosed according to the International Diabetes Federation criteria, was found in 2.3% of Turkish schoolchildren aged 10-19 years. This rate was 28% in obese children. Preventive public measures have started to be implemented by the State and other bodies to control the rising trends in obesity.

**Conflict of interest:**None declared.

## METHODS

Pediatric obesity is a public health problem of increasing importance in the developed world and in populations undergoing cultural and economic transition. As of 2010, global estimates indicate that approximately 1.5 billion adults over 20 years of age are overweight and that more than 200 million men and nearly 300 million women are obese. Nearly 43 million children under the age of 5 years are overweight according to the estimates of the World Health Organization (WHO 2006).

The prevalence of childhood obesity is increasing all over the world, leading to an increase in obesity-related health problems which are expected to have a serious impact on the physical and psychosocial well-being of children in the coming decades as well as on life expectancy, health care costs and national economies. By year 2035, it is estimated that the prevalence of coronary heart disease will increase by 5 to 16%, with more than 100 000 excess cases, attributable to increased obesity among today’s adolescents (1). Adolescents with type 2 diabetes (T2DM) will be at high risk for limb amputation, chronic renal failure requiring dialysis, and premature death. In some, fatty liver will progress to hepatitis and cirrhosis. It is predicted that pediatric obesity may shorten life expectancy by 2 to 5 years by midcentury in the United States (1). 

As a transitional society, obesity figures in Turkey have also shown an increase in the past three decades in both adults and children. Obesity trends of adults in Turkey clearly demonstrate a significant increase, from 16.4% in year 1990 to 32% in year 2000 (2). There is no nationwide systematic study investigating the obesity trends in Turkish children. However, it has been reported that in 6-16-year-old girls living in Istanbul, the overweight+obesity rate was 17.9% in 2001 and increased to 23.4% in 2009 (3). These figures represent a 2-2.5-fold increase as compared to the frequency reported by Neyzi et al (4) in girls in Istanbul in 1966, where 9.4% of the girls were obese or overweight. 

Although there is no large-scale nationwide study on prevalence of childhood obesity in Turkey, numerous papers emerged in the last decade reporting prevalence of childhood obesity in different parts of the country (Table 1). In this review, we tried to present the available information regarding prevalence of overweight and obesity in children and adolescents in Turkey. We selected studies with large numbers of subjects and age range close to the 6-16-year-old age range. In general, lower prevalence rates were reported from the eastern parts of the country; for example, the prevalence rates of overweight and obesity in Van were 11.1% and 2.2%, respectively, and in Elazig - 13.2% and 1.6%, respectively (5,6). Higher figures such as 12.4% and 7.8% in Bursa, 17.6% and 4.3% in Istanbul were obtained in the western parts of the country and in the larger cities (7,8). These differences are most probably related to the relatively lower socioeconomic status of populations in the eastern part of Turkey. It has been shown that obesity is more prevalent in children of higher socioeconomic level in Turkey. This finding is in contrast with findings of studies performed in the USA where obesity is more prevalent among children of lower socioeconomic level. It is known that fresh fruits and vegetables are more expensive than high-calorie processed food in the USA, while the opposite is fortunately still true in Turkey.

**Prevalence of Overweight and Obesity in **

**Different Regions of Turkey **

Findings from different regions in Turkey are given in Table 1. 

The study from Van, an eastern city of Turkey, included 9048 school children aged 6-18 years (5). The study showed that 2.2% of the population in the sample was obese and 11.1% - overweight. The prevalence of obesity was similar in both genders. In boys, the prevalence was extremely low before age 9 and after age 15, but reached high values at puberty and in the peripubertal period. In girls, the peak prevalence was also reached at pubertal ages. In another study from eastern Turkey, Pirincci et al (6) investigated the prevalence of obesity in children aged 6-11 years living in Elazig. A total of 1768 girls and 1860 boys were included in the study (6). The prevalence of obesity and overweight were 1.6% and 13.2%, respectively. In this study, the prevalence of obesity was higher in boys (2.0%) than in girls (1,2). This might be related to the relatively younger age of the subjects since most of them were prepubertal. Pirincci et al (6) also investigated the factors related to obesity and found that parental education, parental body mass index (BMI), family income, and eating habits (e.g. eating while watching TV, eating fast food) all contributed to the development of obesity.

In the Marmara region of Western Anatolia, the prevalence and correlates of obesity were studied in a similar age group from Bursa, the fourth largest city of Turkey. The authors investigated the prevalence of overweight, obesity and severe obesity according to BMI in a total of 5368 children aged 6-12 years. Prevalence figures were 12.4%, 7.8% and 2.2%, respectively (7). The female/male ratio among obese children was 1.24. Eighty percent of obese children had at least one obese parent. Age, gender, presence of obesity in parents, higher educational level of the parents, consumption of soft drinks, physical activity level, and higher income of the family were found to be factors contributing to obesity. The prevalence rates of obesity in Kocaeli and Bolu were 6.8% and 6.1%, respectively (9,10). These figures were similar to that reported from Bursa since these cities are located very close to each other. In Istanbul, the largest city of Turkey, we observed rates of 17.6% for overweight and 4.3% for obesity in 2001 (8). Thus, with the exception of Edirne, a city also in this geographical region, the Marmara region seemed to have the highest prevalence of childhood obesity among the studied regions of Turkey. Oner et al (11) reported data concerning height and weight of 989 adolescents aged 12-17 years and found that obesity prevalence was 2.1% in girls and 1.6% in boys. The prevalence of overweight was 10.6% for girls and 11.3% for boys (11).

In the Aegean region of Western Turkey, Discigil et al (12) screened 1348 children with an age range of 6-16 years in a study from Aydin, a small city in this region. The prevalence rates of obesity and overweight were 3.7% and 12.2%, respectively. High socioeconomic status was found to be associated with childhood obesity. In a large study from Izmir, the third largest city of Turkey, Kalkan Ucar et al (13) investigated 11 629 children aged 2-15 years and found overweight and obesity prevalences of 9.9% and 6.3%, respectively. In the southern city of Antalya, Turkkahraman et al (14) studied 2465 children aged 6-17 years and found that 3.6% of children were obese and 14.3% were overweight. They also studied factors related to obesity. There was no gender difference in obesity prevalence among schoolchildren. In this study, number of regular meals, number of siblings, high birthweight, having a computer at home, skipping breakfast and high socioeconomic status were identified as obesity-related risk factors.

In the north, the West Black Sea region of Turkey, Simsek et al (10) reported that living in an urban setting in a developing country was a risk factor for obesity. In their large cohort, the prevalence rates of obesity in urban and rural areas were 7.7% and 3.9%, respectively (10).

In the Central Anatolia Region, obesity seemed to be less prevalent. In Kayseri, Krassas et al (15) reported a prevalence of 10.6% for overweight and 1.6% for obesity in 2004. In the same year in Ankara, Uckun-Kitapci et al (16) reported prevalence of 10.7% for overweight and 3.6% for obesity.

Obesity prevalence is also on the rise in Turkish children living in Western Europe and the prevalence in this group is higher as compared to Turkish children living in Turkey. This was demonstrated in a study from the Netherlands in which children of multi-ethnic origin were compared. Turkish children had the highest BMI, and when compared to Moroccan children, higher prevalence was found for metabolic syndrome (MS) (22.8% vs. 12.8%), low high-density lipoprotein (HDL)-cholesterol (37.9% vs. 25.8%), hypertension (29.7% vs. 18.0%), and for insulin resistance (54.9% vs. 37.4%) (17). In the same study, it was demonstrated that the prevalence of overweight decreased in Dutch girls from 12.6% to 10.9%, while it increased in Turkish boys from 14.6% to 21.4% from year 1999 through 2007. Figures for prevalence of obesity also increased in Turkish boys and girls from 7.9% to 13.1% and from 8.0% to 10.7%, respectively. Dutch boys, Moroccan and Surinamese South Asian boys and girls showed no similar trends.

**Risk Factors for Childhood Obesity**

Genetic factors, race, sociocultural status, high birthweight, duration of breastfeeding, dysfunctional household, a global shift in the diet, sedentary life, changing modes of transportation and increasing urbanization are factors found to be related with obesity in different studies worldwide (1). In their study on 253 obese children living in Istanbul, Turan et al (8) reported that mothers’ BMI, beverage consumption, sweet-chocolate consumption, activity less than 7 hours/week, and time spent watching TV and playing PC were significantly associated with obesity. Sleep is another risk factor for obesity. Cross-sectional studies indicate relationships between decrease in duration of sleep and obesity and/or insulin resistance. Obesity risk was 2.06-, 1.74-, and 1.86-fold in children who sleep ≤8 hr/d, 8-9 hr/d, and 9-10 hr/d, respectively, as shown by Ozturk et al (18).

The recent advances in technology have dramatically changed the life-style of children in many countries. Fast foods have been the food of choice not only because of time constraints, but also because of the competitive advertisements of the fast food companies. A recent study about the content analysis of food advertising in Turkish TV demonstrated that 32.1% of all TV advertisements were about food, with 81% being about products high in calories, fat and sugar. They were scheduled most commonly for weekends and for mid-afternoon (hours when children watch TV) (19) on weekdays. The same study showed that duration of TV viewing is significantly longer in obese children (2.9±1.2 hours/day vs. 2.3±1.3 hours/day in non-obese) and 89.6% of obese children eat or drink while watching TV.

In summary, high-income family, urban children, obese parents, large birthweight, soft drinks and time spent in front of TV and PC are risk factors identified for childhood obesity from studies performed in Turkey.

**Co-morbidities Associated with Obesity**

The altered nutritional state in obesity results in several alterations that have been linked as co-morbidities of the disease (Table 2). Insulin resistance and T2DM are among the most important health consequences related to obesity. Approximately one-third of obese children and adolescents have insulin resistance (20). Kurtoglu et al (21) examined obese children and adolescents and found insulin resistance in 37% of the boys and 27.8% of the girls before puberty. These frequencies were 61.7% for boys and 66.7% for girls during puberty. When insulin secretion cannot maintain the degree of hyperinsulinemia needed to overcome the resistance, prediabetes (impaired glucose tolerance, impaired fasting glucose) and then T2DM develops. In a multicenter study from Marmara region, the prevalence of prediabetes was 15.2% in obese adolescents and 25.5% in obese children who also had a positive family history of T2DM (22). The frequency of hyperinsulinism was 57.1% in the total group. Prediabetic children had significantly increased levels of homeostasis model of assessment-insulin resistance (HOMA-IR) (11.5+/-7.1). Obesity alone (without family history of T2DM) resulted in 6% of prediabetes in that study.

MS is a term used to describe the clustering of metabolic risk factors for T2DM and atherosclerotic heart disease in adults. The definitions of MS in children differ according to the National Cholesterol Education Program (NCEP), International Diabetes Federation (IDF) and WHO. Thus, the prevalence also varies according to which criteria are used in the analysis. Approximately 10% of US adolescents have MS, as defined according to the adult criteria, modified for age. Cizmecioglu et al (9) reported that 2.3% of Turkish schoolchildren aged 10-19 years had MS when the IDF criteria were applied. They also stated that the prevalence was similar with the NCEP criteria but higher (2.8%) when the WHO definition of MS was used. The prevalence of MS in obese children in this same study was 28.1%, 28.9%, and 34.25% according to the IDF, NCEP and WHO criteria, respectively. In 2010, Budak et al (23) from Kayseri reported that the overall prevalence of MS (modified Adult Treatment Panel (ATP) III criteria) in 12-19-year-old adolescents was 10.8%. In another study by Agirbasli et al (24), the prevalence of MS (ATP III criteria) in 10-17 years children was reported to be 2.2%. The prevalence was 21% in overweight and obese children in the same study (24). In 352 obese children and adolescents, Sen et al (25) reported the prevalence of MS as 41.8%. Younger age of obesity onset, sedentary lifestyle, as well as higher levels of fasting blood glucose, insulin, triglycerides, very-low-density lipoprotein (VLDL) cholesterol, and alanine aminotransferase (ALT) were observed in cases with MS, while the levels of HDL cholesterol and the number of hours spent in physical activity were lower in those subjects (p<0.05). The authors found that the most important determinant of MS was BMI z-score (r=0.31, p<0.0001). A one-point increase in BMI z-score yielded a 2-fold increase in the prevalence of MS. In their study, the prevalence of MS increased from 27.6% to 60.7% when the BMI z-score increased from 2.3 to 3.3.

Obesity is an important risk factor for hypertension and early cardiovascular abnormalities such as atherosclerotic lesions. The incidence of asymptomatic hypertension in school children in Turkey was studied by Akgun et al (26). The incidence of systolic and diastolic hypertension was reported to be 2% and 7%, respectively, in a total of 1963 children in their study. The authors also reported that obesity was present in 10.5% of girls and 13.9% of boys with hypertension. Mazicioglu et al (27) studied 2860 students of whom 246 were hypertensive. BMI, waist circumference (WC) and waist-to-height ratio (WHtR) were reported as significant risk factors for hypertension.

Obesity-related cardiovascular risk was also studied in Turkish children and adolescents. According to Pac et al (28), arterial stiffness may be an indicator of early vascular changes signaling the development of vascular disease. They reported that reduced aortic diastolic velocity is the most prominent early vascular change detected by tissue Doppler imaging in obese children before MS occurs. Obesity in children is associated with arterial wall alterations and endothelial dysfunction. Yilmazer et al (29) showed that carotid intima-media thickness (cIMT) was significantly increased in obese adolescents, whereas carotid artery compliance and brachial artery flow-mediated dilatation were decreased. They also demonstrated that only hypertriglyceridemia was positively correlated with cIMT. The echocardiographic evaluation of epicardial adipose tissue (EAT) thickness has been suggested as an easy method for the evaluation of cardiovascular risk in adults. Ozdemir et al (30) studied this parameter in children and found that EAT thickness correlated significantly with BMI, left atrial diameter, and left ventricular mass. They concluded that there is a close relation between EAT thickness and obesity (30).Nonalcoholic fatty liver disease (NAFLD) is another obesity-related complication which can be asymptomatic, but has potential to progress to hepatitis and even cirrhosis. Eminoglu et al (31) reported that 53% of 101 obese children had NAFLD detected by ultrasonography (USG) and 13.8% of these had elevated ALT levels. They also suggested that BMI and VLDL are the most important determinants of NAFLD, as well as elevated ALT (31). In their study comprising 322 children, Arslan et al (32) reported a frequency of 11.8% for NAFLD. They also found an association between elevated triglyceride levels and fatty liver. According to Ozkol et al (33), elevated BMI was associated with increased risk of fatty liver assessed by B-mode and Doppler USG, and when using Doppler USG, low HDL levels can be used as a good predictor for presence of NAFLD in overweight and obese adolescents. Respiratory function is also affected by obesity. Obstructive sleep apnea describes complete obstruction of upper airway during sleep and cessation of air movement despite ongoing respiratory effort. It may cause right ventricular hypertrophy and may be life-threatening. The prevalence of obstructive sleep apnea was 12.5% in obese children in an Italian study. The prevalence was lower in overweight and normal children - 5.8% and 4.6%, respectively (34). Asthma is also more common in obese children. Exercise test was positive in 31.6% of obese children, whereas it was positive only in 3.3% of healthy controls in the study reported by Ulger et al (35). In this same study, airway hyperresponsiveness, as demonstrated by 4.5% hypertonic saline provocation test, was found to be positive in 18.4% of obese children, and BMI showed strong negative correlations with basal forced vital capacity, forced expiratory volume in 1 second, and peak expiratory flow. All these findings point out the importance of diagnosis and management of exercise-induced bronchospasm in obese children. Treatment of obesity and weight loss may improve exercise performance and physical activity, enhance weight loss, and break the vicious cycle.

It has been demonstrated that there is a strong relationship between BMI and pubertal development in girls. There are many reports from different countries of the world stating that obese girls have earlier breast development and earlier age at menarche. Obese girls reached menarche 10, 9, and 5.5 months earlier than the non-obese ones in Thailand, Japan, and Germany, respectively (36,37,38). In Turkey, Atay et al (39) also reported an earlier age at menarche for obese girls (12.9 vs. 12.5 years). Obese girls are also more prone to develop premature adrenarche, exaggerated adrenarche with precocious puberty and polycystic ovary syndrome. According to Siklar et al (40), prepubertal girls with obesity or insulin resistance are also at risk to develop the full polycystic ovary syndrome phenotype after puberty.

Psychosocial problems related to obesity in children are very important but they can easily be neglected. In community-based studies, obese children are reported to show decreased physical, emotional, social and school performance as compared to their non-obese counterparts. Erermis et al (41) reported that the findings in 16/30 clinically obese adolescents were consistent with a diagnosis of mental disorder, according to the Diagnostic and Statistical Manual of Mental Disorders (DSM-IV), and that this diagnosis often involved a major depressive disorder. The results of this study supported previously published reports showing a high rate of psychopathology (depression, behavioral problems, low self-esteem, etc.) among clinically obese adolescents.

In summary, the prevalence of obesity, although still lower than in North America and Western Europe, is increasing in Turkey in parallel to the trend in many countries. Children living in larger cities and under better economic conditions seem to be at higher risk. Complications and co-morbidities of obesity have also started to appear in our pediatric population, a warning that efforts should focus on prevention of obesity during childhood. Importance should be given to programs targeting healthy nutrition and activities in schools. Public measures such as regulations on food advertising on TV and food labelling are also needed and relevant programs should be started as rapidly as possible and be conducted all over the country. It is encouraging to witness that such programs are being implemented by the joint activities of the Turkish Pediatric Endocrinology Society and both the Ministries of Health and Education. Recently, the first steps were taken for regulations on high-calorie food advertising targeting children on TV and regulations on food sold in school canteens. A new initiative called DOMATES (Diabetes and Obesity Management at the School), launched in 2011, and aiming to improve diabetes care in schools will be elaborated to include healthy eating habits and exercising in schools. 

## Figures and Tables

**Table 1:**
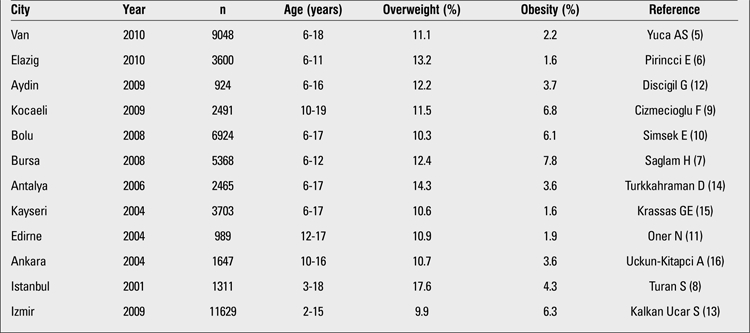
Prevalence of childhood overweight and obesity in different cities of Turkey

**Table 2:**
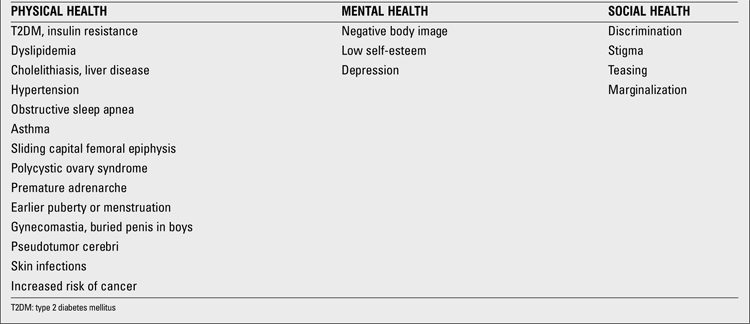
Co-morbidities associated with obesity
